# ADAM17/PTGS2 Facilitates Pulmonary Fibrosis by Regulating Ferroptosis

**DOI:** 10.1111/jcmm.70466

**Published:** 2025-03-12

**Authors:** Suyan Yan, Yaqi Zhao, Wei Xu, Jin Zhang, Ying Zhang, Baocheng Liu, Xinya Li, Zhenzhen Ma, Qingrui Yang

**Affiliations:** ^1^ Department of Rheumatology and Immunology Shandong Provincial Hospital Affiliated to Shandong First Medical University Jinan China; ^2^ Department of Rheumatology and Immunology Shandong Provincial Hospital, Cheeloo College of Medicine, Shandong University Jinan Shandong China; ^3^ Shandong University of Traditional Chinese Medicine Jinan Shandong China

**Keywords:** ADAM17, ferroptosis, fibroblasts, PTGS2, pulmonary fibrosis

## Abstract

Pulmonary fibrosis (PF) is a chronic and progressive interstitial lung disease characterised by excessive deposition of extracellular matrix (ECM), resulting in high mortality rates. In this study, we provide evidence that ADAM17/PTGS2 plays a crucial role in inducing ferroptosis in fibroblasts, promoting PF. Initially, an assessment was made of ADAM17 protein levels in patients diagnosed with connective tissue diseases–interstitial lung diseases (CTD‐ILD), using ELISA assays. Confirmation of the relationship between ADAM17 and fibrosis was achieved by stimulating cells with PMA or TAPI‐1 (the ADAM17 inhibitor), in conjunction with the fibrosis‐inducing factor, TGFβ1. To further explore the major downstream proteins of ADAM17 contributing to altered PF, we employed mRNA transcriptomics. To further investigate the role of ADAM17/PTGS2 in promoting ferroptosis and fibrosis, we employed western blot assays, immunofluorescence and transmission electron microscopy (TEM). Furthermore, the effects of the ADAM17/PTGS2/ferroptosis pathway in PF were verified using Adeno‐associated virus (AAV)‐mediated ADAM17 gene knockdown in mice. In CTD‐ILD patients, ADAM17 expression was significantly elevated. Upon PMA stimulation, lung fibroblasts exhibited increased fibrosis‐related proteins, and the combined stimulation of PMA and TGFβ1 synergistically promoted cellular fibrosis. Conversely, TAPI‐1 alleviated fibrotic stimulation induced by TGFβ1. Transcriptomic analysis of lung fibroblast specimens overexpressing ADAM17 revealed significantly elevated PTGS2 expression levels. Knockdown and ferroptosis inhibition assays demonstrated that ADAM17 regulates ferroptosis in lung fibroblasts via PTGS2, ultimately inducing fibrosis. Furthermore, the deficiency of ADAM17 alleviated bleomycin‐induced PF and inflammation in mice. These findings first verified that ADAM17/PTGS2/ferroptosis is a novel mechanism for regulating PF; it provides a new theoretical basis for further exploring the treatment of PF.

**Abbreviations**
ACE2: angiotensin‐converting enzyme 2
ACSL4: acyl‐CoA synthetase long‐chain family member 4
ADAM17: A disintegrin and metalloprotease 17
Ang II: angiotensin II
BLM: bleomycin
CTD‐ILD: connective tissue disease‐associated interstitial lung disease
ECM: extracellular matrix
EGFR: epidermal growth factor receptor
ERK: extracellular signal‐regulated kinase
Fer‐1: Ferrostatin‐1
GPX4: Glutathione peroxidase 4
IL‐1β: interleukin‐1β
IL‐6: interleukin‐6
PGE2: prostaglandin E2
PMA: Phorbol 12‐myristate 13‐acetate
PTGS2: prostaglandin G/H synthase 2
TGFβ1: transforming growth factor β1
TNF‐α: tumour necrosis factor α
α‐SMA: alpha‐smooth muscle actin

## 1 Background

Connective tissue diseases (CTD) are systemic conditions characterised by autoimmune overactivation. Interstitial lung diseases (ILD) affect approximately 40%–50% of CTD patients [1] and represent a common pulmonary complication of CTD. ILD is characterised by lung parenchymal damage caused by varying degrees of inflammation and fibrosis, which finally result in abnormal lung remodelling and repair. Such fibrosis impairs alveolar ventilation, causing dyspnoea and significantly contributes to the mortality rate among CTD patients [2, 3]. While significant progress has been made in the treatment of ILD with the discovery of immunosuppressive agents and antifibrotic drugs such as nintedanib and pirfenidone [2, 4], currently available drugs offer limited remission of forced vital capacity (FVC), and the prognosis and quality of life for patients have not notably improved. Therefore, further exploration of the pathogenesis of ILD and the discovery of promising therapeutic targets to prevent PF development remain crucial tasks.

A disintegrin and metalloproteinases (ADAM17) is a membrane protein with proteolytic cleaving enzyme activity anchored to cell membranes. ADAM17 plays a vital physiological role in cell adhesion, proliferation and nervous system development [5, 6, 7], as well as in pathological processes such as inflammatory responses, tumour progression and metastasis [8, 9]. In the respiratory tissues, ADAM17's enzymatic substrates, including epidermal growth factor receptor (EGFR) ligands, MMPs, CX3XL1 and tumour necrosis factor‐α (TNF‐α), are involved in the cellular inflammation and fibrosis processes [10, 11, 12, 13]. However, the intracellular molecular mechanism through which ADAM17 induces lung fibrosis remains unclear, necessitating further studies to elucidate how ADAM17 promotes lung fibrosis development and progression.

In our study, mRNA transcriptome sequencing analysis revealed elevated prostaglandin G/H synthase 2 (PTGS2) levels in lung fibroblasts following the upregulation of ADAM17 expression. PTGS2 is a major regulator of ferroptosis, a novel form of regulated cell death driven by iron‐dependent lipid peroxidation and influenced by various factors and mechanisms, including iron, lipid, amino acid metabolism and mitochondrial activity [14, 15]. Emerging evidence suggests that ferroptosis is implicated in fibrotic diseases [16], where it contributes to fibrosis progression by inducing inflammation and parenchymal cell death [17]. Our study demonstrates that ADAM17 regulates ferroptosis in lung fibroblasts to promote fibrosis development through PTGS2, suggesting a potential therapeutic target in ADAM17 for the treatment of lung fibrosis.

## 2 Material and Methods

### 2.1 Human Subjects

Serum samples were collected from patients diagnosed with CTD at the Department of Rheumatology and Immunology, Shandong Provincial Hospital affiliated with Shandong First Medical University from March 2017 to December 2021. The diagnosis of PM/DM was based on the established international criteria of Bohan and Peter in 1975 [18, 19]; RA was based on the American College of Rheumatology/European League Against Rheumatism (ACR/EULAR) classification criteria in 2010 [20]; pSS was based on the ACR classification criteria in 2012 [21]; SLE was based on the Systemic Lupus International Collaborating Clinics (SLICC) criteria for SLE in 2012 [22]; SSc was based on the ACR/EULAR classification criteria in 2013 [23]. The diagnosis of ILD was based on typical high‐resolution computed tomography scan findings [24]. Patient information is provided in Table S1. Patients who had chronic or acute infection, metabolic diseases, chronic liver or kidney diseases, various malignancies, poisoning, chronic obstructive pulmonary disease and other diseases were excluded from this study. All patients included in the study were sampled before receiving systemic medication. Control samples were randomly selected from the medical examination centre for a health checkup, and they had no evidence of illness during the same period. This study was approved by the Ethics Committee of Shandong Provincial Hospital (NSFC: NO. 2022–413), and written informed consent was obtained from all participants.

### 2.2 Cell Culture and Treatment

Human fibroblasts (MRC‐5 cells) were procured from ATCC and cultured in minimum essential medium (Gibco, USA) supplemented with 10% foetal bovine serum (Gibco, USA) at 37°C with 5% CO_2_. Fibroblast activation was induced by treating cells with 10 ng/mL recombinant TGFβ1 (Sino Biological, China) for 48 h. To modulate ADAM17 expression, fibroblasts were exposed to 25 ng/mL PMA (Solarbio, China) or 1 μM TAPI‐1 (MedChemExpress, China) for 48 h.

ADAM17 plasmids, PTGS2 siRNA and control siRNA were synthesised by Keyybio (Shanghai, China). Cell transfections were performed using the Lipofectamine 2000 reagent (Thermo Fisher Scientific, USA) following the manufacturer's instructions. The sequences for PTGS2 siRNA and ADAM17 plasmid are provided in Table S2. The ferroptosis inhibitor Fer‐1 was purchased from MedChemExpress.

### 2.3 Construction of the Animal Model

Male C57BL/6 mice (6–8 weeks old, 20–25 g) were acquired from HFK Bioscience (Beijing, China) and were housed in a temperature‐controlled environment with a 12‐h light and dark cycle. Mice had unrestricted access to food and water. All animal experiments adhered to the guidelines for the Care and Use of Experimental Animals of Shandong Province Hospital and received approval from the Animal Experiment Ethics Committee of Shandong Provincial Hospital affiliated with Shandong First Medical University (NSFC: NO. 2022–413).

To manipulate ADAM17 expression in the lungs, mice were intratracheally instilled with an adeno‐associated serotype 9 (AAV9‐U6‐shRNA‐CMV‐GFP) viral vector carrying mouse ADAM17 shRNA (AAV‐shADAM17) or control (AAV‐SC) (Genomeditech, China). The infection dose consisted of AAV diluted in 50‐μL saline to a final concentration of 1.5 × 10^11^ v.g AAV‐shADAM17. The sequences for ADAM17 shRNA are provided in Table S3.

PF models were established in mice 1 week after AAV infection. Mice were anaesthetised with 1% pentobarbital sodium and then administered 2.5 mg/kg body weight of BLM in 50‐μL normal saline through intratracheal injection. Control mice received an equivalent volume of saline. All mice were euthanised after 21 days. Bronchoalveolar lavage fluid (BALF) was collected through bronchoalveolar lavage with 0.6 mL of sterile phosphate‐buffered saline (PBS), as previously described [25].

### 2.4 Histological Staining

The upper left lung lobe was excised, fixed in 4% paraformaldehyde for 48 h, and then embedded in paraffin. Tissue sections, 5‐μm‐thick slices, were prepared for histological staining. The sections were deparaffinised, rehydrated and stained with haematoxylin–eosin (H&E), Masson's trichrome reagents and Sirius red staining, following the manufacturer's instructions. For immunohistochemistry (IHC), tissue sections were subjected to antigen retrieval by immersion in 0.01 M sodium citrate and heated at 95°C for 15 min. This was followed by incubation with 3% hydrogen peroxide at room temperature for 10 min to deactivate endogenous peroxidase, and then blocking with 5% bovine serum albumin (BSA). Tissue sections were incubated overnight at 4°C with anti‐ADAM17 (1:500 dilution) antibodies. After PBS wash, sections were incubated with antirabbit (IHC kit) secondary antibodies at 37°C for 1 h. Staining was visualised using a DAB substrate kit, and histological images were captured using a Pannoramic Scanning Electron Microscope (3DHISTECH). Quantification was performed using ImageJ software.

### 2.5 Immunofluorescence Assay

The bottom left lung was removed, fixed in 4% paraformaldehyde for 48 h, embedded in the OCT compound and then frozen. Tissue sections, 5‐μm‐thick slices, were obtained and fixed with cold acetone for 10 min. Subsequently, they were incubated with 0.5% Triton X‐100 for 30 min to permeabilise the cell membrane. Tissues were blocked with 2% goat serum and then incubated overnight at 4°C with anticollagen I (1:300 dilution), anti‐PTGS2 (1:100 dilution) and anti‐ADAM17 (1:100 dilution) antibodies. Following this, tissue sections were incubated with fluorescence‐conjugated secondary antibodies for 1 h at 37°C in the dark. Cell nuclei were stained with DAPI, and fluorescent images were captured using a fluorescence microscope (Olympus, Tokyo, Japan).

### 2.6 Transwell Migration Assay

MRC‐5 cells were trypsinised, resuspended in serum‐free minimal essential medium and seeded into cell culture inserts with 8‐μm pore size. These inserts were incubated with MRC‐5 conditional medium for 24 h, exposing the cells to various stimuli. Afterwards, we removed the cells from the upper side of the membrane, while the cells on the bottom membrane were fixed and stained using a crystal violet solution. We then captured images of the migrated cells with a light microscope and counted them in five fields.

### 2.7 Glutathione (GSH) and Oxidised Glutathione Disulfide (GSSG) Levels

We utilised the GSH and GSSG Assay Kit (Beyotime, China) to measure the concentrations of GSH and GSSG in differently pretreated MRC‐5 cells following the manufacturer's protocol.

### 2.8 TEM

MRC‐5 cells were seeded onto six‐well chambered cover glass (Thermo Fisher Scientific) at a density of 2 × 10 ^ 5^4^ cells/mL (14,000 cells/well). Images were captured using the Olympus EM208S transmission electron microscope.

### 2.9 Assessment of Intracellular ROS

The production of intracellular free ROS was separately detected using DHE (10 μM, Beyotime, China), following the manufacturer's recommendations. After staining, we immediately acquired images in the dark using a fluorescence microscope (Olympus, Tokyo, Japan), and the fluorescence intensity of the probe was evaluated using ImageJ software.

### 2.10 Enzyme‐Linked Immunosorbent Assay (ELISA)

We determined the BALF contents of IL‐1β and TNF‐α using their respective ELISA kits, following the manufacturer's instructions. Briefly, each well of the microplate was precoated with 100 μL of diluted capture antibodies overnight, followed by blocking with 1% BSA. Next, 100 μL of the sample or standard substance was added to each well and incubated for 2 h. This was followed by the addition of 100 μL of detection antibodies per well. After that, samples were incubated with streptavidin–horseradish peroxidase (HRP)–conjugated secondary antibodies for 30 min, followed by incubation with a detection substrate. Lastly, 50 μL of 2 N H_2_SO_4_ was added to stop the reaction, and the sample absorbance was measured using a microplate reader at 450 nm.

### 2.11 Real‐Time Fluorescence Quantitative Polymerase Chain Reaction (RT‐qPCR)

RNA was isolated from cells or tissues using TRIzol (Invitrogen, USA) following standard protocols, and cDNA was synthesised using the PrimeScript RT reagent Kit (Takara, China). Quantitative real‐time PCR was performed with the TB Green Premix Ex Taq II (Takara) on the SYBR Green I/HRM Dye PCR System (Roche 480II). Data were normalised to a housekeeping gene (GAPDH for human samples) and analysed using the 2^−△△Ct^ method relative to saline/vehicle‐treated control groups. The data are derived from at least three independent experiments performed in triplicate, and the RT‐qPCR primers used are presented in Table S4.

### 2.12 Western Blot

Total protein was extracted from cells or crushed tissues using lysis buffer (150 mM NaCl, 10 mM Tris pH 7.2, 5 mM EDTA, 0.1% sodium dodecyl sulphate (SDS), 1% sodium deoxycholate, 1% Triton X‐100 and protease inhibitors). Sample protein concentrations were determined using a bicinchoninic acid protein assay kit and adjusted for immunoblotting analysis. A total of 30 μg of proteins was separated by SDS–polyacrylamide gel electrophoresis and transferred onto polyvinylidene difluoride membranes. The membranes were blocked with 5% BSA in Tris‐buffered saline with Tween 20, followed by overnight incubation with primary antibodies at 4°C. Subsequently, the membranes were incubated with HRP‐coupled secondary antibodies, and the results were visualised using ECL luminescent liquid on a Tanon 5200 Multi FluorChem imaging system.

The following antibodies used and their manufacturers: the antibody against collagen I (ABclonal, China), the antibody against α‐SMA (Abcam, USA), the antibody against ADAM17 (Abcam), the antibody against PTGS2 (Abcam), the antibody against ACSL4 (Abcam), the antibody against GPX4 (Abcam) and the antibody against GAPDH (ABclonal).

### 2.13 Statistical Analysis

The data are presented as mean ± standard error of the mean (SEM). Statistical analyses were performed using GraphPad Prism 9.0 (GraphPad Software, USA). Differences in quantitative data between the two groups were determined using Student's two‐tailed t‐test. One‐way analysis of variance (ANOVA), followed by post hoc multiple comparisons, was used for comparing more than two groups. Two‐way ANOVA (Tukey post hoc test) was performed to compare multiple groups. In all cases, a significance level of *p* < 0.05 was defined as statistically significant, with *, # and % indicating *p* < 0.05, and **, ## and %% indicating *p* < 0.01.

## 3 Results

### 3.1 Upregulation of ADAM17 in TGFβ1‐Stimulated Fibroblasts and Fibrotic Lungs

**FIGURE 1:**
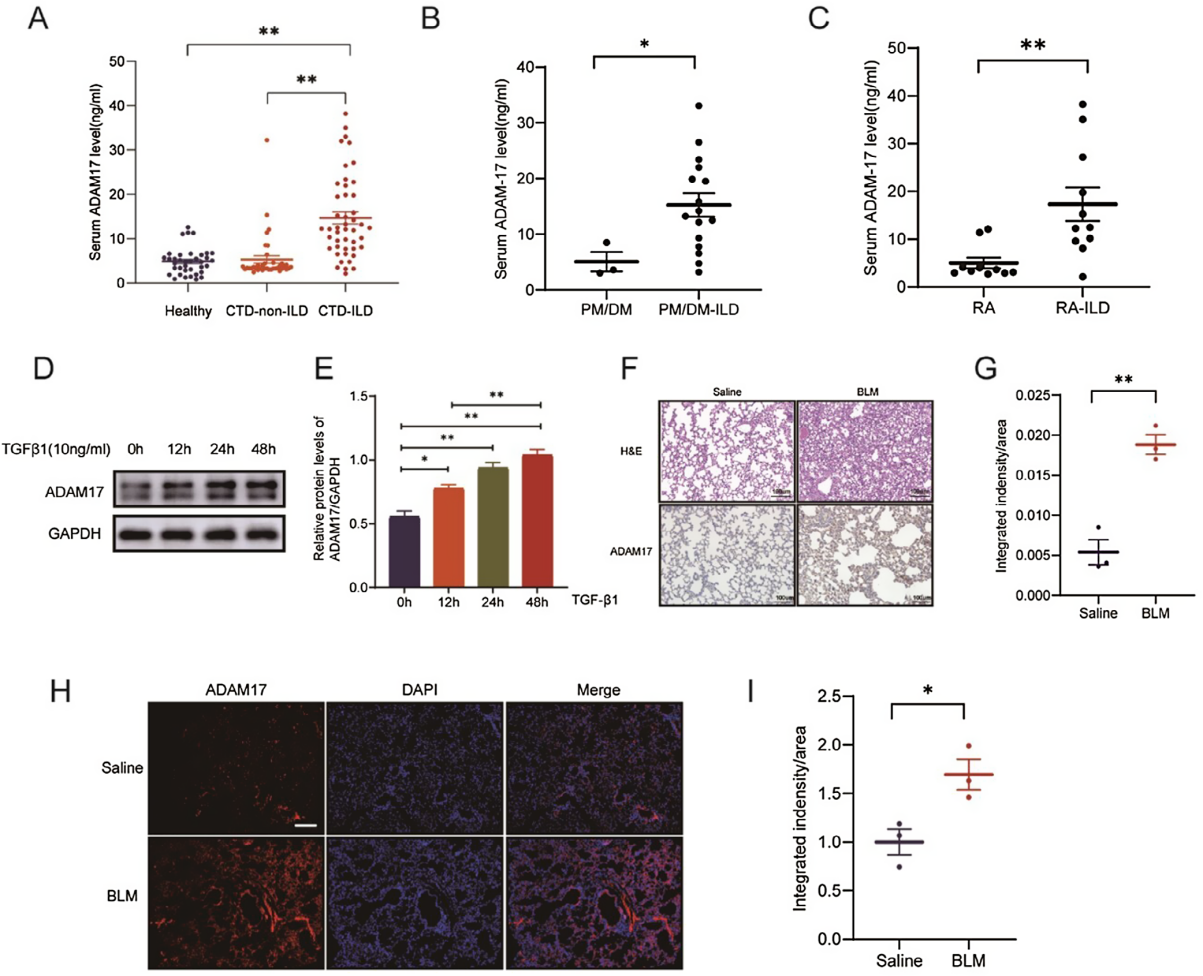
ADAM17 is overexpressed in TGFβ1‐stimulated fibroblasts and fibrotic lungs. (A) Serum expression levels of ADAM17 in connective tissue disease (CTD) patients with or without Interstitial lung disease (ILD). (B) Serum expression levels of ADAM17 in PM/DM patients with or without ILD. (C) Serum expression levels of ADAM17 in RA patients with or without ILD. (D) Serum expression levels of ADAM17 in pSS patients with or without ILD. (E‐F) Western blot and corresponding densitometry analysis of ADAM17 in TGFβ1‐treated (10 ng/mL for 0, 12, 24 and 48 h) MRC‐5 cells. (G and H) Immunohistochemistry staining of ADAM17 in MRC‐5 cells. (I) Representative results of ADAM17 immunofluorescence staining in lung tissue sections from BLM‐induced lung fibrosis mice. Data are presented as the mean ± SEM wherein the statistical analyses included unpaired Student's *t*‐tests, one‐way analysis of variance (Tukey post hoc test) as appropriate. The data presented were taken from three independent experiments; scale bar = 100 μm, *p < 0.05, **p < 0.01.

First, we collected clinical samples for enzyme‐linked immunosorbent assay (ELISA) testing from 121 individuals, including 37 healthy individuals, 38 CTD‐non‐ILD and 46 CTD‐ILD. All diagnosed patients belonged to the initial diagnosis and treatment group. We found that ADAM17 was highly expressed in patients with CTD‐ILD (Figure 1A). Further analysis of ADAM17's differential expression by rheumatic disease types revealed statistically significant expression in PM/DM and RA with ILD patients, but not in pSS with ILD patients (Figure 1B–D). To elucidate ADAM17's specific role in ILD, we examined its expression distribution in lung tissues using the human protein atlas. We found that, apart from macrophages, ADAM17 was predominantly expressed in lung fibroblasts and epithelial cell lines (https://v22.proteinatlas.org/ENSG00000151694‐ADAM17/single+cell+type). Consequently, we examined ADAM17 expression after inducing fibroblasts and lung epithelial cells with TGFβ1 (10 ng/mL) for varying durations in vitro. As expected, ADAM17 increased in a time‐responsive manner in human embryonic lung fibroblasts (MRC‐5 cells; Figure 1E,F). However, no significant difference was observed in ADAM17 expression over time in lung epithelial cells (Figure S1A,B). Immunohistochemical analysis demonstrated high ADAM17 expression in BLM‐induced fibrotic lung tissues (Figure 1G,H). Additionally, immunofluorescence staining confirmed high ADAM17 expression in BLM‐induced mouse lung tissues (Figure 1I). These data support ADAM17 activation in the fibrotic environment, suggesting its crucial role in regulating pulmonary fibro‐degeneration.

### 3.2 Upregulation of ADAM17 Induces Lung Fibroblast Activation

**FIGURE 2:**
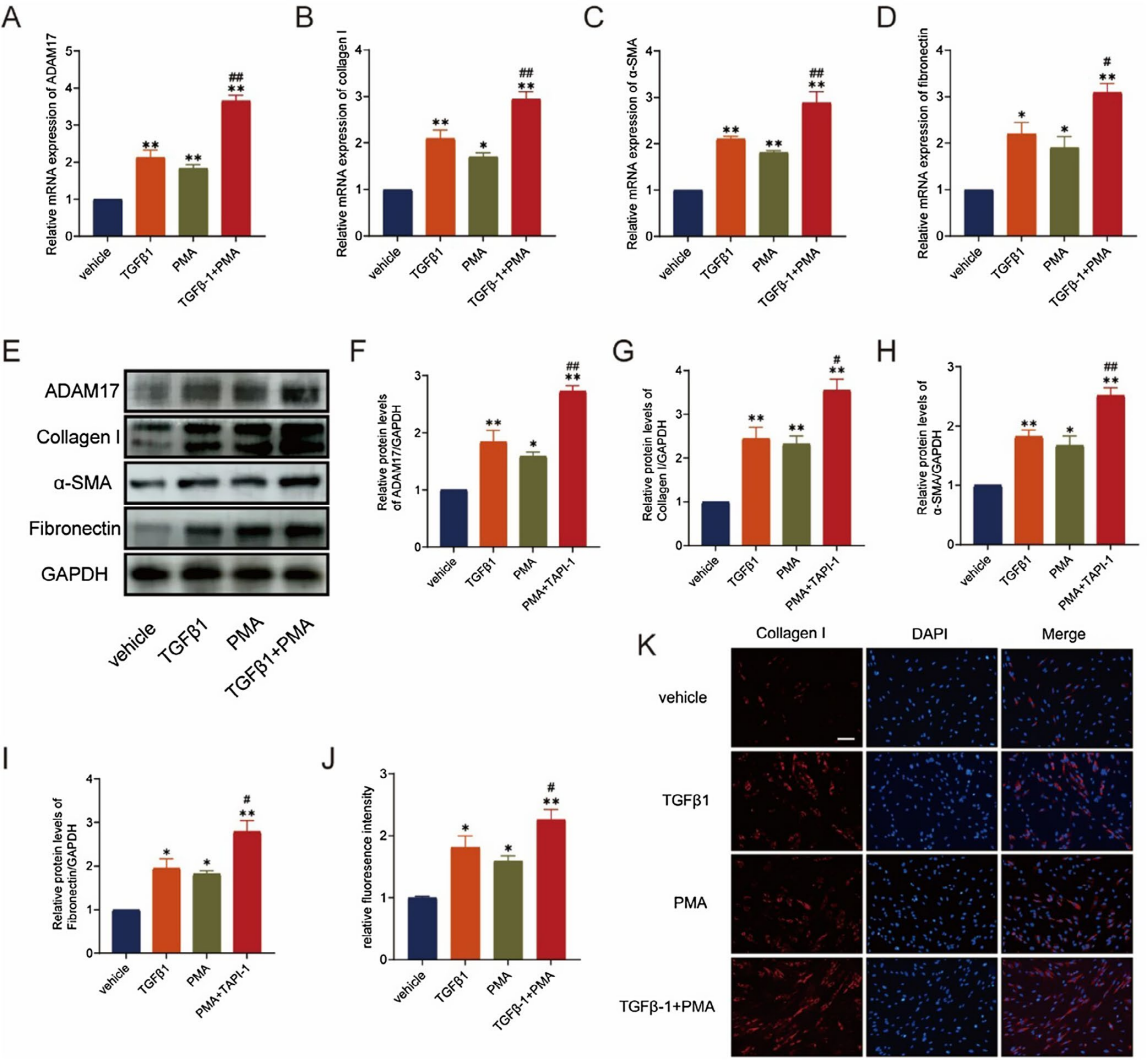
Upregulation of ADAM17 induces lung fibroblast activation. (A and F) RT‐qPCR and western blot analysis of ADAM17 expression in MRC‐5 cells stimulated with phorbol 12‐myristate 13‐acetate (PMA; 100 nM) or TGFβ1(10 ng/mL) for 48 h. (B–E and G–I) RT‐qPCR and western blot analysis of fibronectin, collagen I and α‐SMA in MRC‐5 cells stimulated with PMA or 10 ng/mL TGFβ1 for 48 h. (J) Mean fluorescence intensity of collagen I in MRC‐5 cells from different groups. (K) Expression of collagen I was detected by immunofluorescence staining in MRC‐5 cells stimulated with PMA or TGFβ1 for 48 h. Data are presented as the mean ± SEM, with statistical analysis performed by one‐way ANOVA (Tukey post hoc test) as appropriate. The data presented are taken from three independent experiments; scale bar = 100 μm, *p < 0.05, **p < 0.01 versus vehicle group; #*p* < 0.05, ##*p* < 0.01 versus PMA group.

Although ILD exists with some heterogeneity in histopathologic types, PF is its ultimate manifestation and pulmonary fibroblast activation is a vital link in the formation of PF [2]. The transformation of lung fibroblasts into myofibroblasts is a critical progression in the pathogenesis of PF characterised by excessive production and accumulation of ECM, including α‐SMA, collagen I and fibronectin [26]. Thus, we conducted cell function experiments using the ADAM17 agonist PMA with TGFβ1 as a positive control to investigate whether ADAM17 is necessary for fibroblast activation. Initially, PMA and TGFβ1 significantly upregulated ADAM17 mRNA and protein expression levels in fibroblasts. There was a synergistic effect on ADAM17 mRNA and protein expression in lung fibroblasts after co‐stimulation with PMA and TGFβ1 (Figure 2A,F). Subsequently, elevated levels of fibronectin, collagen I and α‐SMA proteins were observed following ADAM17 upregulation (Figure 2B–E and G–I). Moreover, ADAM17 upregulation promoted collagen I expression, as indicated by collagen I staining in lung fibroblasts (Figure 2J,K). Cell migration assays revealed enhanced migration of lung fibroblasts after ADAM17 activation (Figure 2L,M). These results suggest that ADAM17 facilitates fibrosis by promoting cell migration and the synthesis of fibrosis‐associated proteins, thus activating fibroblast‐to‐myofibroblast transformation.

### 3.3 Inhibition of ADAM17 Arrests Lung Fibroblast Activation

**FIGURE 3:**
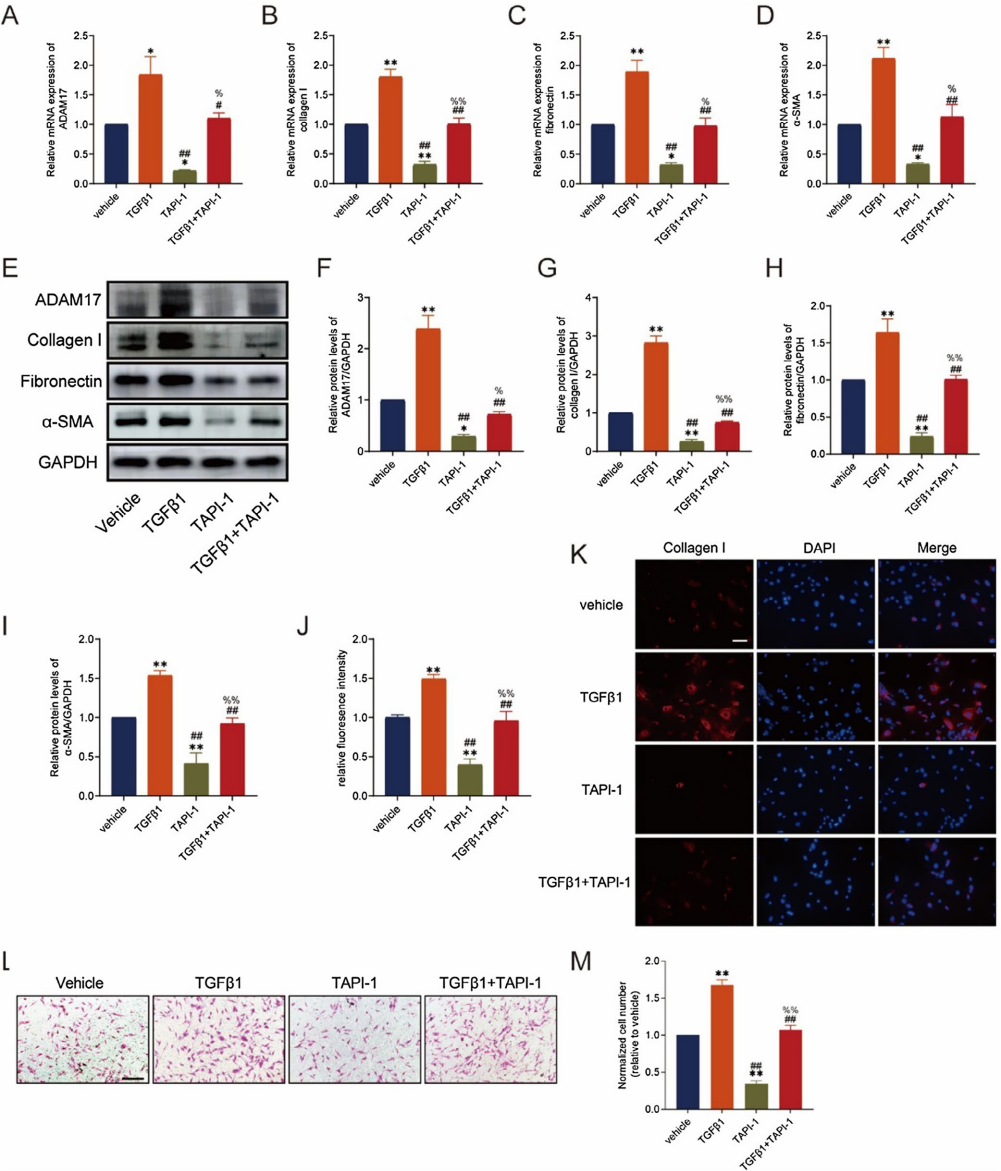
Downregulation of ADAM17 alleviates fibrosis in lung fibroblasts. (A and F) RT‐qPCR and western blot analysis of ADAM17 expression in MRC‐5 cells stimulated with TAPI‐1 or TGFβ1 for 48 h. (B–E and G–I) RT‐qPCR and western blot analysis of fibronectin, collagen I and α‐SMA in MRC‐5 cells stimulated with TAPI‐1 or 10 ng/mL TGFβ1 for 48 h. (J) Mean fluorescence intensity of collagen I in MRC‐5 cells from the different groups. (K) Collagen I expression was detected by immunofluorescence staining in MRC‐5 cells stimulated with TAPI‐1 or TGFβ1 for 48 h. (L) Transwell migration assay showing TGFβ1‐induced and TAPI‐1‐induced cell migration in MRC‐5 cells. (M) The number of migrated cells was counted, and the data were presented in a graph. Data are presented as the mean ± SEM, wherein statistical analysis included one‐way ANOVA (Tukey post hoc test) as appropriate. The data presented are taken from three independent experiments; scale bar = 100 μm. *p < 0.05, **p < 0.01 versus vehicle group; #*p* < 0.05, ##*p* < 0.01 versus TGFβ1 group; %*p* < 0.05, %%*p* < 0.01 versus TAPI‐1 group.

To further confirm ADAM17's activating effect on fibroblasts, we conducted loss‐of‐function experiments using the ADAM17 inhibitor TAPI‐1, with TGFβ1 as a positive control. Initially, TGFβ1 significantly upregulated ADAM17 mRNA and protein expression levels in fibroblasts. TAPI‐1 downregulated ADAM17 mRNA and protein expression in fibroblasts, with TAPI‐1 reversing the upregulation effect on ADAM17 mRNA and protein expression in lung fibroblasts stimulated by TGFβ1 (Figure 3A,F). Subsequently, an increase in fibronectin, collagen I and α‐SMA mRNA and protein levels was observed following ADAM17 upregulation, with a reversal of the increases in fibrotic mRNA and protein levels induced by TGFβ1 after ADAM17 downregulation (Figure 3B–E and G–I). It suggests that inhibition of ADAM17 has a protective effect on fibrosis. Additionally, ADAM17 downregulation inhibited collagen I expression, as evidenced by collagen I staining in lung fibroblasts (Figure 3J,K). Cell migration assays showed diminished lung fibroblast migration after ADAM17 inhibition (Figure 3L,M). Overall, these findings suggest that inhibiting ADAM17 alleviates cell migration and fibrosis‐associated protein synthesis, thereby attenuating fibroblast‐to‐myofibroblast transformation.

### 3.4 Overexpression of ADAM17 Induces PTGS2 Activation in Lung Fibroblasts

**FIGURE 4:**
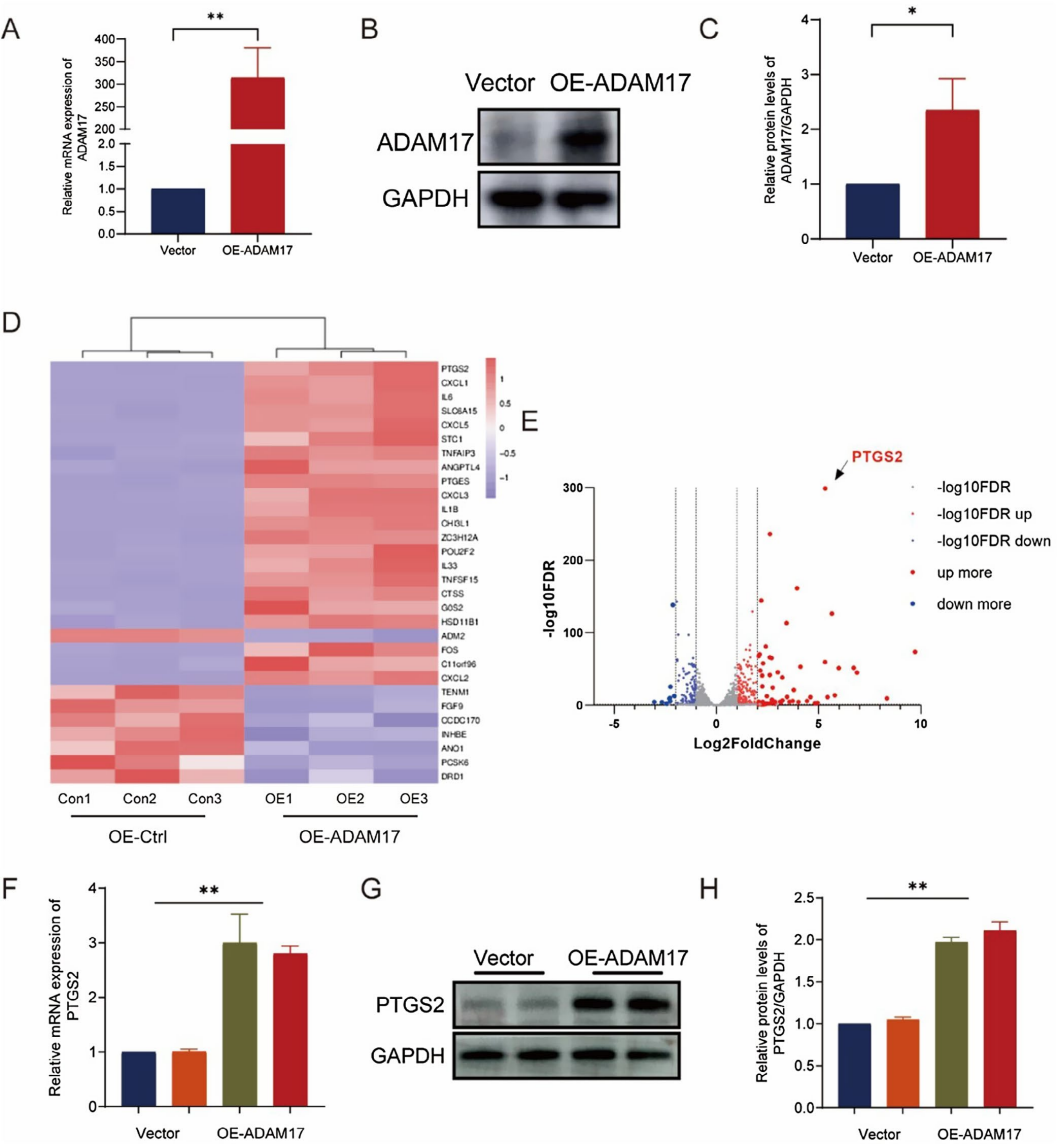
Overexpression of ADAM17 induces PTGS2 activation in lung fibroblasts. (A–C) RT‐qPCR and western blot analyses of ADAM17 expression in MRC‐5 cells transfected with ADAM17 cDNA (OE‐ADAM17) and its negative control cDNA (vector). (D and E) RNA‐sequencing analyses of total RNA from MRC‐5 cells with ADAM17 cDNA (OE‐ADAM17) and its negative control cDNA (vector); the 30 differentially expressed genes are selected with fold change > 2 and false discovery rate < 0.05 between OE‐ADAM17 versus vector. (F‐H) RT‐qPCR and western blot analyses of PTGS2 in MRC‐5 cells transfected with OE‐ADAM17 and vector. Data are presented as the mean ± SEM, with statistical analysis performed using unpaired Student's *t*‐tests as appropriate. The data presented are taken from three independent experiments. *p < 0.05, **p < 0.01.

We have demonstrated the fibrogenic effect of ADAM17 on fibroblasts, but the specific intracellular regulatory mechanism is unclear. To further dissect the regulatory mechanism of ADAM17 in fibroblast signalling, we initially transfected MRC‐5 cells with an ADAM17 plasmid (OE‐ADAM17) and a control plasmid (vector) for 24 h and assessed the RNA and protein expression levels of ADAM17 (Figure 4A–C). Subsequently, MRC5 cells treated with OE‐ADAM17 and Vector underwent RNA sequencing. We selected 30 differentially expressed genes (DEGs), comprising 22 upregulated genes and 8 downregulated genes, with the largest differences between groups based on fold change (FC > 2) and FDR < 0.05 criteria. This suggests that the upregulation of PTGS2 may influence fibrotic signalling in fibroblasts (Figure 4D,E). Additionally, we examined the RNA and protein expression levels of PTGS2 in ADAM17 plasmid (OE‐ADAM17) and control plasmid (vector)‐treated MRC5 cells (Figure 4F–H) and found that PTGS2 was upregulated following the upregulation of ADAM17 expression, indicating a potential role of PTGS2 in regulating fibrotic signalling.

### 3.5 ADAM17/PTGS2 Promotes Lung Fibrosis by Activating Ferroptosis in Lung Fibroblasts

**FIGURE 5:**
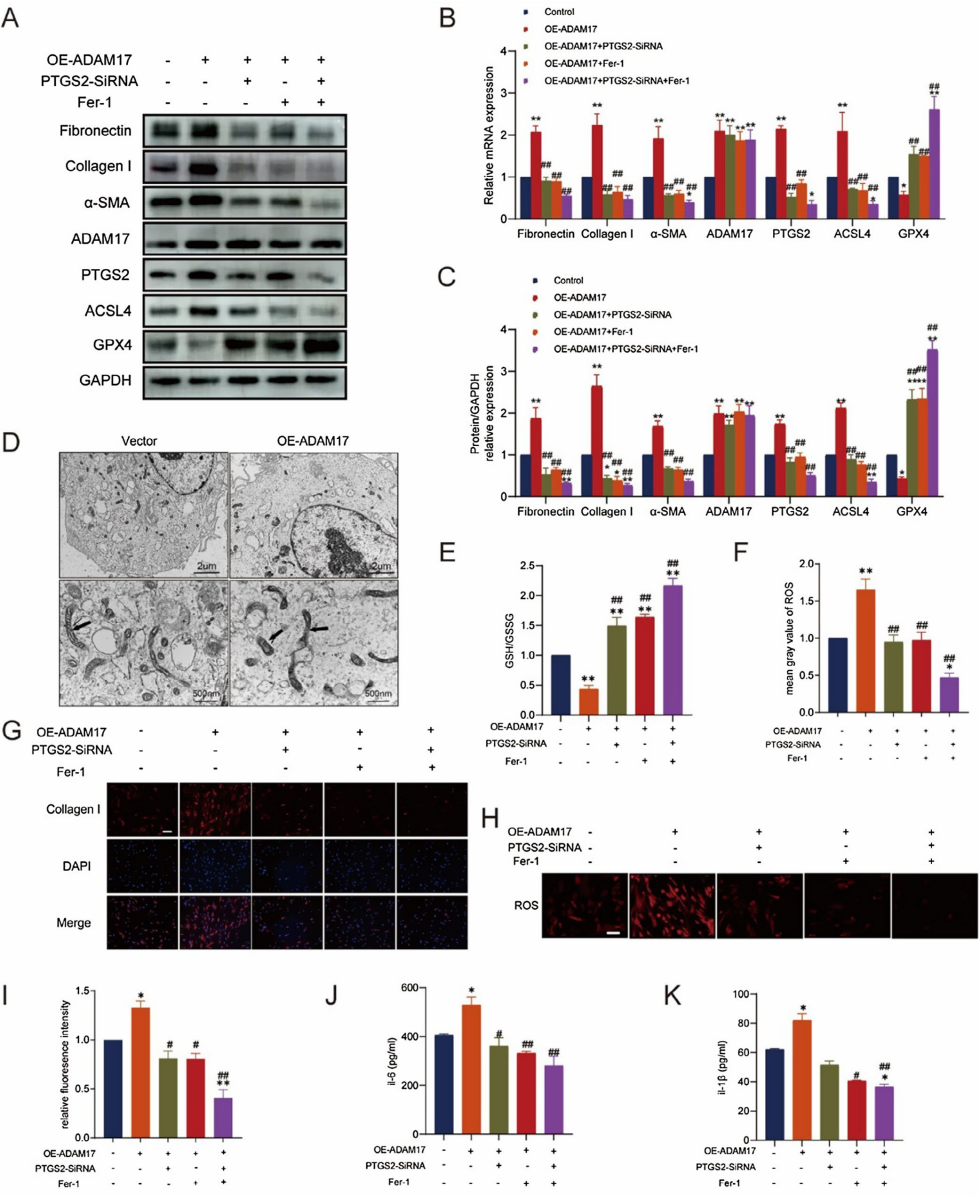
ADAM17 regulates intracellular ferroptosis through PTGS2 to induce fibrosis. (A–C) RT‐qPCR and western blot analysis of fibronectin, collagen I and α‐SMA, ADAM17, PTGS2, ACSL4 and GPX4 expression in MRC‐5 cells transfected with ADAM17 cDNA (OE‐ADAM17), PTGS2‐siRNA, and treated with Fer‐1. (D) Representative TEM ultrastructural photomicrographs showing the mitochondrial structure wherein ferroptosis occurs. (E) Normalised levels of GSH/GSSG were detected in MRC‐5 cells. (F and H) Representative images of staining for reactive oxygen species (ROS) were detected using the fluorescent probe DHE: red, oxidised form of DHE. (G and I) Expression of collagen I was detected by immunofluorescence staining in MRC‐5 cells transfected with ADAM17 cDNA (OE‐ADAM17), PTGS2‐siRNA, and treated with Fer‐1. (J‐K) IL‐6 and IL‐1β in the supernatant of MRC‐5 cells transfected with ADAM17 cDNA (OE‐ADAM17), PTGS2‐siRNA, and treated with Fer‐1, as assessed by ELISA. Data are presented as the mean ± SEM wherein statistical analysis included using one‐way ANOVA (Tukey post hoc test) and two‐way ANOVA (Tukey post hoc test) as appropriate. The data presented are taken from four independent experiments; scale bar = 200 μm. *p < 0.05, **p < 0.01 versus control group; ^#^ *p* < 0.05, ^##^ *p* < 0.01 versus OE‐ADAM17 group.

We observed that ADAM17 overexpression led to elevated PTGS2 expression in lung fibroblasts. Notably, PTGS2 induces disease by mediating ferroptosis, which has been reported in cardiovascular diseases [27]. A growing number of studies have shown that PTGS2 is a key regulator of cellular ferroptosis [28]. Therefore, we hypothesised that ADAM17/PTGS2‐mediated fibrosis is associated with ferroptosis. Using PTGS2‐siRNA, OE‐ADAM17 and Fer‐1 (a ferroptosis inhibitor) to treat MRC‐5 cells, the relevant expression of PTGS2 protein was significantly reduced in the PTGS2 siRNA group compared to the control group. The result is shown in Figure S2. We noted that overexpression of ADAM17 increased the content of ferroptosis‐related indexes in fibroblasts in the OE‐ADAM17 group, and inhibition of intracellular ferroptosis in the OE‐ADAM17 + Fer‐1 group alleviated the level of ferroptosis caused by ADAM17 overexpression, suggesting that ADAM17 promotes ferroptosis in lung fibroblasts. In the OE‐ADAM17 and the OE‐ADAM17 + PTGS2‐siRNA groups, inhibition of PTGS2 reversed the level of ferroptosis induced by ADAM17 overexpression, suggesting that ADAM17 regulates ferroptosis through PTGS2. In the OE‐ADAM17, OE‐ADAM17 + PTGS2‐siRNA group, OE‐ADAM17 + PTGS2‐siRNA and OE‐ADAM17 + PTGS2‐siRNA + Fer‐1 group, we found that inhibition of PTGS2 and ferroptosis reversed the level of fibrosis caused by the overexpression of ADAM17, suggesting that ADAM17 may regulate fibrosis through the regulation of the level of intracellular ferroptosis in lung fibroblasts by PTGS2(Figure 5A–C). TEM analysis revealed that fibroblasts in the ADAM17 overexpression group exhibited a more severe level of ferroptosis compared with the vector group, characterised by the disappearance of intracellular mitochondrial cristae and an increase in mitochondrial density (Figure 5D). GSH/GSSG levels were used to assess antioxidant activity in cells (Figure 5E). ROS measurement suggested varying degrees of ferroptosis in fibroblasts (Figure 5F,H). Immunofluorescence analysis showed that collagen I was upregulated in ADAM17 overexpression and significantly reduced after ferroptosis inhibition treatment (Figure 5G–I). ELISA of cell culture supernatants revealed that the secretion of inflammatory cytokines (interleukin [IL]‐6 and IL‐1β) increased in ADAM17 overexpression and significantly decreased after exposure to PTGS2‐siRNA and Fer‐1 treatment (Figure 5J,K). These results suggest that ADAM17/PTGS2 promotes fibroblast fibrosis by activating ferroptosis in lung fibroblasts.

### 3.6 Intratracheal Instillation of AAV‐shADAM17 Attenuates BLM‐Induced PF and Ferroptosis in Mice

**FIGURE 6:**
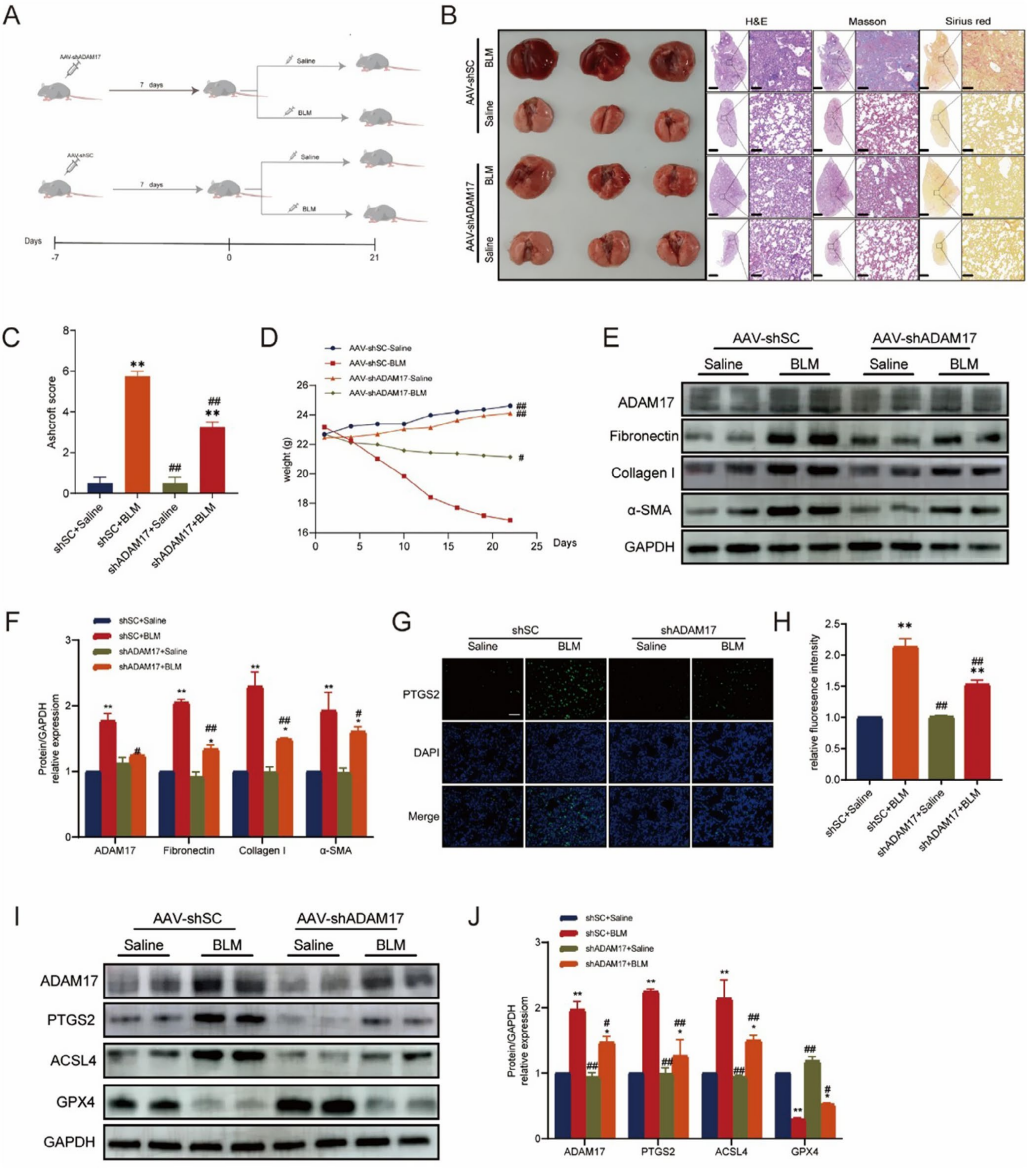
Administration of ADAM17‐shRNA viruses attenuates BLM‐induced lung fibrosis in mice. (A) Schematic showing the procedure of animal experiments. (B) H&E, Sirius red and Masson's trichrome staining assays were performed to measure lung fibrosis severity. (C) Semiquantitative Ashcroft scores indicate fibrosis severity. (D) Weight of different groups of mice during BLM‐induced PF. (E and F) Western blot of ADAM17, fibronectin, collagen I and α‐SMA in mouse lung tissues. (G and H) Immunofluorescence staining of PTGS2 in mouse lung tissues for the indicated groups. PTGS2 shows a green stain; DAPI shows a blue stain. (I‐J) Western blot of ADAM17, PTGS2, ACSL4 and GPX4 in mouse lung tissues. Data are presented as the mean ± SEM, wherein statistical analysis included one‐way ANOVA (Tukey post hoc test) and two‐way ANOVA (Tukey post hoc test) as appropriate. The data presented are taken from three independent experiments, *n* = 4 per group; Scale bar = 100 μm. *p < 0.05, **p < 0.01 versus shSC + saline group; #*p* < 0.05, ##*p* < 0.01 versus shSC + BLM group.

We recognise that the PF model induced by BLM differs in pathology and pathogenesis from CTDs‐ILD, in contrast to IPF. Nevertheless, among the various experimental PF models currently used, BLM induction stands out as the most frequently employed approach [29, 30]. It has been demonstrated that multiple cell types, including type I alveolar epithelial cells, fibroblasts, myofibroblasts, macrophages, lymphocytes, neutrophils, endothelial cells, pericytes, airway epithelial cells and stem/progenitor cells, are implicated in the development of BLM‐induced fibrosis [31]. Therefore, we chose to utilise the BLM‐induced lung fibrosis model in our experiment. Mice were categorised into the following groups: AAV‐shADAM17 + Saline, AAV‐shSC + saline, AAV‐shADAM17 + BLM and AAV‐shSC + BLM, to assess the therapeutic effectiveness of ADAM17‐shRNA in BLM‐induced fibrosis (Figure 6A). Mice transfected with the ADAM17‐shRNA virus exhibited a significant reduction in BLM‐induced ferroptosis and lung fibrosis, as evidenced by H&E, Sirius red, Masson trichrome staining and Ashcroft score (Figure 6B,C). Body weight changes in mice indicated that ADAM17‐shRNA‐transfected mice experienced less weight loss induced by BLM (Figure 6D). Consistently, western blot assays revealed that levels of fibronectin, collagen I, α‐SMA, ADAM17, PTGS2 and ACSL4 in lung tissues from AAV‐shADAM17 mice following BLM induction were lower than those observed in AAV‐shSC mice, in contrast to the expression of GPX4 (Figure 6E,F,I and J). However, in the AAV‐shADAM17 group of mice, the interference with ADAM17 was not statistically significant compared to the control group, probably due to the low initial expression of ADAM17 in the mice, resulting in a lower interference efficiency. An immunofluorescence assay also confirmed that mice transfected with the ADAM17‐shRNA virus significantly reduced PTGS2 expression (Figure 6G,H). These findings suggest that ADAM17 silencing mitigates ferroptosis and alleviates BLM‐induced lung fibrosis.

### 3.7 Intratracheal Instillation of AAV‐shADAM17 Alleviated Inflammation in Experimental Mouse Models

**FIGURE 7:**
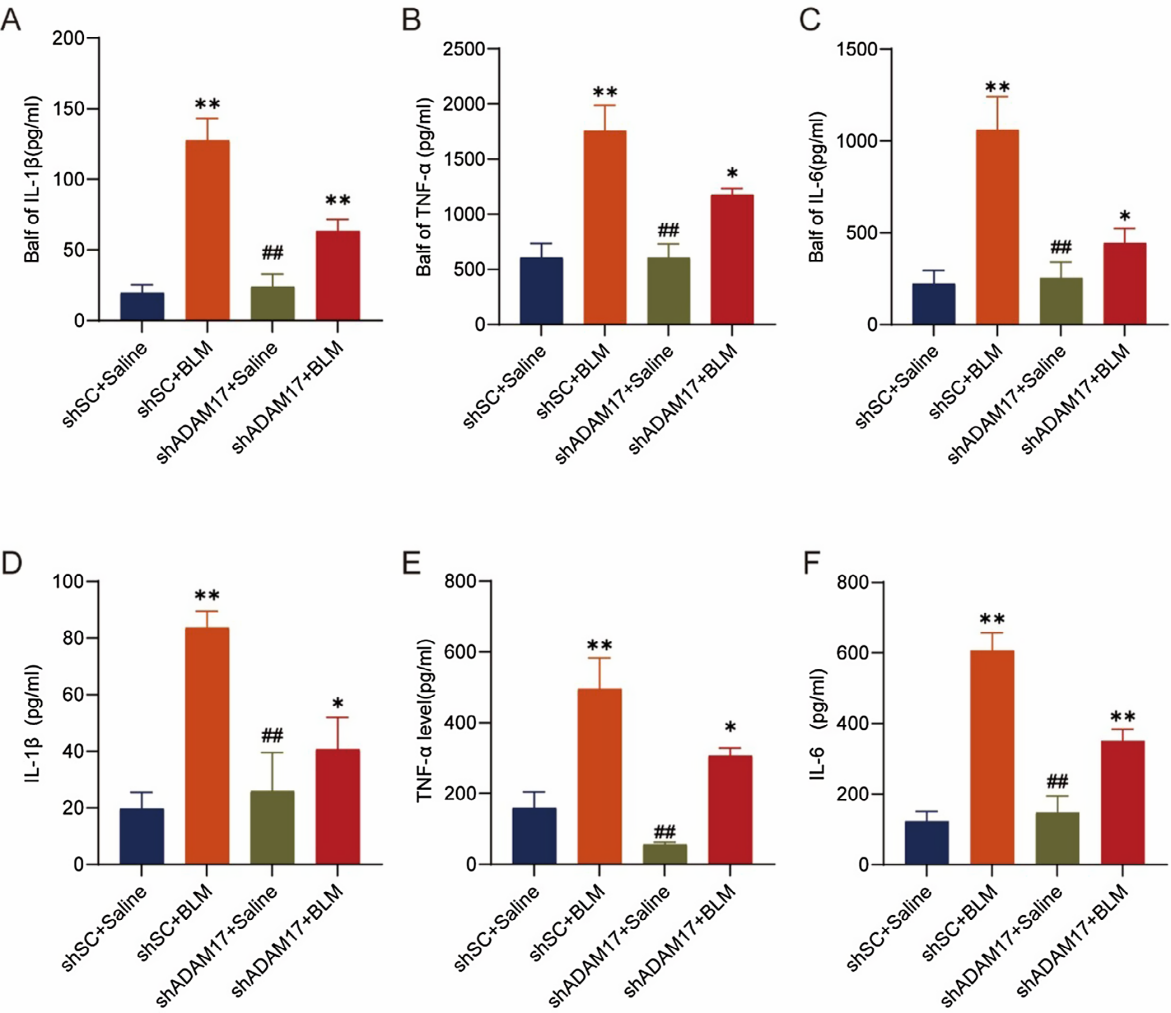
Administration of ADAM17‐shRNA viruses attenuates BLM‐induced inflammation. (A–C) The expression of TNF‐α, IL‐6 and IL‐1β in Balf of mice. (D–F) The expression of TNF‐α, IL‐6 and IL‐1β in peripheral blood of mice. Data are presented as the mean ± SEM, wherein statistical analysis included one‐way ANOVA (Tukey post hoc test) as appropriate. The data presented are taken from three independent experiments, *n* = 4 per group. *p < 0.05, **p < 0.01versus shSC + Saline group; #*p* < 0.05, ##*p* < 0.01versus shSC + BLM group.

Inflammation within tissues is an important trigger for fibrosis development [32]; inflammation is characterised by elevated levels of cytokines such as IL‐1β, TNF‐α and IL‐6. ADAM17 impacts the biology of IL‐1β, TNF‐α and IL‐6. Studies have shown that ADAM17 acts as a substrate‐cleaving enzyme that cleaves IL‐1β, TNF‐α and IL‐6, affecting its activity and thus influencing the further occurrence of intracellular signalling pathways [33]. In our study, we wondered how ADAM17 affects the inflammatory factors in fibrotic mice. Therefore, we detected inflammatory factors in mice by assessing alveolar lavage fluid and peripheral blood from BLM‐induced mice with AAV‐shADAM17 or AAV‐shSC, and the results showed a significant reduction in IL‐1β, TNF‐α and IL‐6 in BLM‐induced AAV‐shADAM17 mice compared with BLM‐induced AAV‐NG mice (Figure 7A–F).

## 4 Discussion

PF is a prevalent complication of CTD, leading to a gradual decline in lung function due to the progressive deposition of ECM, proliferation of fibroblasts and destruction of lung tissue structure. The management of PF remains a significant medical challenge. This study sheds light on the crucial role of ADAM17 in regulating fibroblast function. Key findings include: (1) Significant upregulation of ADAM17 in patients, TGFβ1‐stimulated lung fibroblasts and fibrotic mouse lungs. (2) Upregulation of ADAM17 expression promotes lung fibroblast activation; downregulation of ADAM17 expression ameliorates TGFβ1‐induced lung fibroblast activation. (3) ADAM17 overexpression drives pulmonary fibrogenesis by initiating intracellular ferroptosis via PTGS2. (4) Intratracheal injection of an ADAM17 shRNA‐carrying virus reverses BLM‐induced lung fibrotic degeneration and ferroptosis in mice. These findings highlight the ADAM17/PTGS2/ferroptosis pathway as a key mechanism in the pathogenesis of pulmonary fibrotic degeneration, suggesting that targeted therapy against ADAM17 may be a feasible clinical approach for the treatment of pulmonary fibrotic degeneration.

The identification of ADAM17 as the TNF‐α converting enzyme has revealed a plethora of insights into protein ectodomain shedding, significantly impacting cell development and biological functions [34, 35]. Furthermore, dysregulation of ADAM17 is strongly linked to numerous common pathological conditions, such as asthma, arthritis, cancer and fibrosis [36]. Prior research has illustrated that ADAM17 can induce fibrosis and liver regeneration by activating EGFR ligands, initiating the intracellular RAF/MEK1/2/ERK1/2 signalling pathway [37, 38]. Additionally, ADAM17 cleaves cell membrane IL‐6R, releasing soluble IL‐6R, which binds to IL‐6 forming complexes that act on gp130 protein on immune cell membranes, activating the intracellular JAK/STAT pathway to induce renal fibrosis [39, 40, 41]. In cardiac fibrosis, ADAM17 releases ACEII into the extracellular fluid through cleavage, where soluble ACEII converts Ang II to Ang1‐7, unlike the pro‐inflammatory and proliferative properties of Ang II [42, 43] mitigating myocardial fibrosis, myocardial hypertrophy and cardiomyocyte apoptosis by binding to MRSR [44]. In this study, we observed elevated ADAM17 expression in the serum of patients with CTD combined with ILD. Additionally, we found that fibroblast activation and excessive ECM production can contribute to pulmonary fibrotic degeneration. These findings indicate a harmful effect of ADAM17 on CTD‐ILD, necessitating further investigation into its mechanism of action in fibrotic lung disease. To explore the fibrogenic role of ADAM17 at the cellular and molecular level, we examined its function in fibroblasts in vitro. First, we established ADAM17 overexpression in TGFβ1‐induced fibroblasts and fibrotic mouse lung tissue. Stimulation of fibroblasts with the ADAM17 agonist PMA resulted in fibroblast activation and elevated intracellular fibrosis‐related markers (collagen I, fibronectin and α‐SMA). Loss‐of‐function studies demonstrated that inhibition of ADAM17 reduced fibroblast activation and reversed TGFβ1‐induced fibroblast activation, prompting a focus on ADAM17's mechanism in fibroblasts.

Prostaglandins (PGs) are cyclooxygenase (COX)‐dependent arachidonic acid metabolites that play crucial roles in inflammatory responses. PGE2, PGD2, 15d‐PGJ2 and PGI2 mainly mediate antifibrotic reactions by binding to their homologous receptors and activating downstream G protein‐related kinase reactions along with the induction of cAMP synthesis, leading to the inhibition of proliferation and migration, transformation of interstitial cells such as fibroblasts into myofibroblasts and the promotion of apoptosis to reduce the deposition of ECM proteins in various organs [45]. Moreover, these PGs inhibit the apoptosis of parenchymal cells, thereby reducing organ structural failure and dysfunction [46]. However, PGE2 and PGD2 also play a role in promoting fibrosis in certain tissues and cells [47]. These contradictory findings are related to the type of receptors, EP1/EP3, and the cells studied [48]. However, PGF2α and TXA2 exert opposite effects compared to the aforementioned PGs and mainly promote the occurrence of fibrotic reactions [49]. Through sorting, we found that the level of PGs changes in different disease states. For example, in patients with IPF, the levels of COX‐2/PGE2 in alveolar lavage fluid are decreased, and the level of PGF2α is increased [50]. Although these changes in PGs are different, the combined effect of these changes contributes to the development of fibrosis in patients.

In lungs, prostacyclin [51, 52, 53] has been shown to inhibit activation and proliferation of lung fibroblasts in vitro and in vivo. PGF2α is identified as an important mediator of pulmonary fibrosis by enhancing proliferation and collagen synthesis of lung fibroblasts through F‐prostanoid receptor in a TGF‐β‐independent manner [54]. Furthermore, Kida et al. found that PGD2 plays a protective role in bleomycin‐induced lung inflammation and pulmonary fibrosis [55]. However, PGs, especially those induced by COX‐2 (like PGE2), are most closely related to the pathogenesis of pulmonary fibrosis.

PTGS2, a key enzyme in PGE2 synthesis, is considered one of the key factors regulating ferroptosis [56]. The induction of disease by PTGS2 through mediating ferroptosis has been well reported in cardiovascular diseases [57]. Ferroptosis is a nontraditional form of programmed cell death characterised by iron overload and lipid peroxidation [14]. Ferroptosis has received increasing attention in the field due to the accumulation of reactive oxygen species and excessive iron deposition leading to the development of a variety of lung diseases. Recent evidence suggests that the activation of ferroptosis is involved in the process of lung fibrosis, while inhibiting ferroptosis could have antifibrotic effects [58]. Studies have shown that reducing iron concentration and accumulation attenuates lung fibrotic degeneration in a BLM mouse model [59]. Moreover, the ubiquitin‐like containing PHD and RING finger domains 1‐regulated ferroptosis inhibits the GPX4 and FSP1 genes, promoting lung fibrotic degeneration [60]. Ferroptosis also influences fibrosis by modulating the transformation of epithelial‐mesenchymal cells [61]. These findings indicate a regulatory effect of ferroptosis on lung fibrosis. In our study, we observed an elevation in PTGS2 expression in fibroblasts transfected with the ADAM17 plasmid, as evidenced by transcriptome sequencing analysis. PTGS2, a well‐known regulator of ferroptosis, appears to play a pivotal role in this process. Previous studies have favoured that PTGS2‐ferroptosis‐associated pulmonary fibrosis is through an inflammatory response: Ferroptosis is associated with increased expression of PTGS2, which leads to increased secretion of PGE2, accelerated metabolism of arachidonic acid and release of inflammatory mediators, which activate the inflammatory response and lead to pulmonary fibrosis [62]. However, it is reasonable to infer that ferroptosis itself more likely represents a downstream pathway in ADAM17/PTGS2‐induced lung fibrosis during fibroblast activation. Encouragingly, our subsequent experimentation involved treating fibroblasts transfected with the ADAM17 plasmid with PTGS2‐siRNA and Fer‐1 ferroptosis inhibitor. Inhibition of ferroptosis resulted in reduced expression of intracellular iron metamorphosis markers (e.g., ROS and ACSL4), along with increased levels of GSH/GSSG and GPX4, suggesting that ADAM17 could regulate the reduction of ferroptosis through PTGS2. In addition, treatment of lung fibroblasts transfected with the ADAM17 plasmid by PTGS2‐siRNA suppressed fibrosis‐related markers, including collagen I, fibronectin and α‐SMA, thereby reducing fibrosis progression. This result is consistent with the use of Fer‐1 (a ferroptosis inhibitor), which also attenuated fibrosis in lung fibroblasts. In animal model experiments, we demonstrated that adeno‐associated virus‐based interference with lung ADAM17 significantly attenuated the BLM‐induced PF and ferroptosis in mice. The above results propose that ADAM17 upregulates intracellular ferroptosis by enhancing PTGS2 expression, thereby promoting PF progression.

While previous studies have primarily focused on ADAM17's role in activating intracellular cascade pathways or inflammatory factors to induce fibrosis [12, 63], our research identifies a new mechanism of ADAM17/PTGS2 mediating PF through ferroptosis. In addition, there are some shortcomings in our study. The results were biased due to the small number of serum samples from CTD‐ILD patients, but the elevated expression of ADAM17 in ILD patients is not the first time it has been confirmed in this study, and more studies have reported the same findings [64, 65], which increased the credibility of the results. Notably, PTGS2, a key enzyme in prostaglandin E2 (PGE2) synthesis, primarily initiates various inflammatory factors [66]. Although it serves as a marker protein for ferroptosis, its direct involvement in ferroptosis is indirect, likely through the regulation of autophagy or the PTGS2/PGE2 pathway. Therefore, the mechanism through which ADAM17/PTGS2 is indirectly involved in ferroptosis needs to be further explored.

## 5 Conclusions

We verified that ADAM17 is elevated in fibrotic lung disease from three perspectives: clinical patients and in vitro and in vivo studies; and our in vivo and in vitro studies provide the specific mechanisms by which ADAM17 activates PF. We demonstrated for the first time that ADAM17/PTGS2 facilitates pulmonary fibrogenesis by initiating intracellular ferroptosis and proposed that ADAM17/PTGS2/ferroptosis is a new pathway driving PF, providing a new theoretical basis for further exploration of PF treatment.

## Author Contributions

**Suyan Yan:** formal analysis (equal), writing – original draft (equal). **Yaqi Zhao:** data curation (equal), software (equal). **Wei Xu:** investigation (equal). **Jin Zhang:** software (equal). **Ying Zhang:** resources (equal). **Baocheng Liu:** methodology (equal). **Xinya Li:** project administration (equal). **Zhenzhen Ma:** writing – review and editing (equal). **Qingrui Yang:** funding acquisition (equal), writing – review and editing (equal).

## Ethics Statement

Ethics approval for the study protocol was obtained from the Shandong Provincial Hospital affiliated with Shandong First Medical University. All participants in this study provided informed consent in accordance with guidelines set by the ethical committee (NSFC: NO. 2022–413).

## Consent

All authors have reviewed the final version of the manuscript and approved it for publication.

## Conflicts of Interest

The authors declare no conflicts of interest.

## Supporting information


**Figure S1.** (A and B) Western blot and corresponding densitometry analysis of ADAM17 in TGFβ1‐treated (10 ng/mL for 0, 12, 24 and 48 h) lung epithelial cells. Data are presented as the mean ± SEM wherein statistical analysis is included using one‐way ANOVA (Tukey post hoc test) as appropriate.
**Figure S2.** (C and D) Western blot analysis of PTGS2 in MRC‐5cells transfected with PTGS2‐siRNA and its control group. Data are presented as the mean ± SEM wherein statistical analysis is included using one‐way ANOVA (Tukey post hoc test) as appropriate. *p < 0.05, **P < 0.01 versus control group.
**Table S1.** Serological and general information on patients with CTD‐ILD.
**Table S2.** The siRNA sequences targeting PTGS2.
**Table S3.** The shRNA sequences targeting ADAM17.
**Table S4.** Primer sequence (human).

## Data Availability

The data that support the findings of this study are available from the corresponding author upon reasonable request.
