# Supplementary material for: ADAM17/PTGS2 Facilitates Pulmonary Fibrosis by Regulating Ferroptosis
Source: J Cell Mol Med. 2025 Mar 12;29(5):e70466. doi: 10.1111/jcmm.70466 (PMC11903495; doi:10.1111/jcmm.70466)
Supplement: Supplementary file 1 — Figure S1. (A and B) Western blot and corresponding densitometry analysis of ADAM17 in TGFβ1‐treated (10 ng/mL for 0, 12, 24 and 48 h) lung epithelial cells. Data are presented as the mean ± SEM wherein statistical analysis is included using one‐way ANOVA (Tukey post hoc test) as appropriate. Figure S2. (C and D) Western blot analysis of PTGS2 in MRC‐5cells transfected with PTGS2‐siRNA and its control group. Data are presented as the mean ± SEM wherein statistical analysis is included using one‐way ANOVA (Tukey post hoc test) as appropriate. *p < 0.05, **P < 0.01 versus control group. Table S1. Serological and general information on patients with CTD‐ILD. Table S2. The siRNA sequences targeting PTGS2. Table S3. The shRNA sequences targeting ADAM17. Table S4. Primer sequence (human). [file JCMM-29-e70466-s001.docx]

**Supplementary Figure 1.**


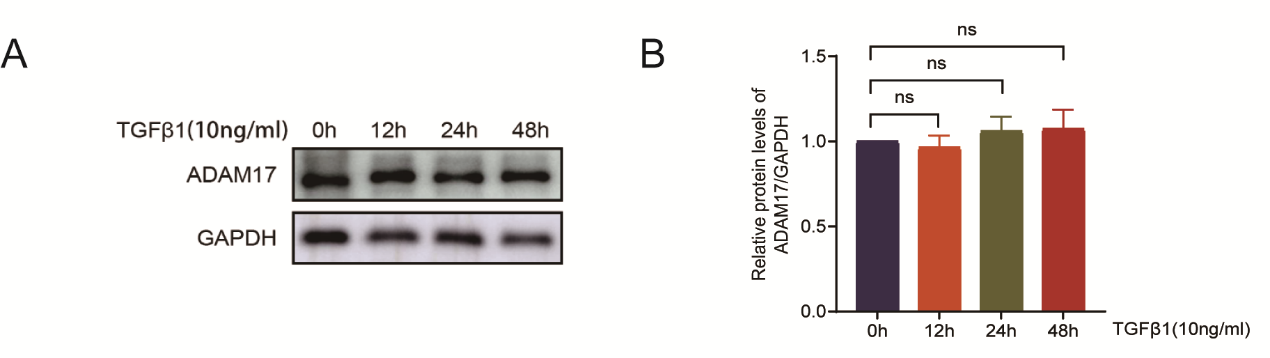


(**A-B**) Western blot and corresponding densitometry analysis of ADAM17 in TGFβ1-treated (10 ng/mL for 0, 12, 24, and 48 hours) lung epithelial cells. Data are presented as the mean ± SEM wherein statistical analysis included using one-way ANOVA (Tukey post hoc test) as appropriate.

**Supplementary Figure 2.**


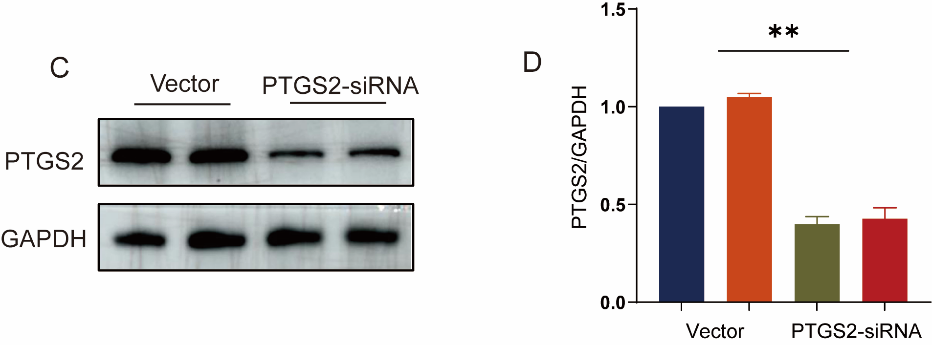


(**C-D**) Western blot analysis of PTGS2 in MRC-5cells transfected with PTGS2-siRNA and its control group. Data are presented as the mean ± SEM wherein statistical analysis included using one-way ANOVA (Tukey post hoc test) as appropriate. *P<0.05, **P＜0.01 versus control group.

**Supplementary Table 1.**

**Serological and general information on patients with CTD-ILD.**

|  | Age | Gender | Smoking | Drinking | ADAM17 level |
| --- | --- | --- | --- | --- | --- |
| SSc-ILD | 50.86±3.48 | 6 (86%) | 1 (14.3%) | 1(14.3%) | 10.29±2.3 |
| RA-ILD | 66.5±2.33 | 6 (54.5%) | 2 (18.2%) | 3（27.2%） | 18.08±3.75 |
| SLE-ILD | 52.14±5.43 | 5 (71.4%) | 2 (28.6%) | 1(14.3%) | 13.64±3.82 |
| pSS-ILD | 53.6±2.16 | 5 (100%) | 0 | 0 | 14.75±4.66 |
| PM/DM-ILD | 58.63±2.47 | 8 (50%) | 5 (31.2%) | 3(18.8%) | 15.25±2. 08 |
| RA | 53.38±3.93 | 9 (90%) | 2 (20%) | 1(12.5%) | 3.5 (2.72-12.1) |
| SLE | 33.1±3.98 | 11 (100%) | 0 | 0 | 3.4 (2.47-8.57) |
| pSS | 60.92±3.85 | 14 (100%) | 0 | 1(7.1%) | 3.43 (3.18-32.27) |
| PM/DM | 53±11.36 | 3 (100%) | 0 | 0 | 5.07±1.73 |

ADAM17: A disintegrin and metalloprotease 17; CTD-ILD: Connective tissue disease-associated interstitial lung disease; SSc: Systematic sclerosis; RA: Rheumatoid arthritis; SLE: Systemic Lupus Erythematosus; pSS: Primary Sjogren's Syndrome; PM/DM: Polymyositis and Dermatomyositis.

**Supplementary Table 2.**

**The siRNA sequences targeting PTGS2**

| PTGS2（human）siRNA-1683 | UGAAAGGACUUAUGGGUAATT |
| --- | --- |
|  | UUACCCAUAAGUCCUUUCATT |
| PTGS2（human）siRNA-612 | CAAAUGAGAUUGUGGAAAATT |
|  | UUUUCCACAAUCUCAUUUGTT |
| PTGS2（human）siRNA-270 | GGACAGGAUUCUAUGGAGATT |
|  | UCUCCAUAGAAUCCUGUCCTT |
| Control | UUCUCCGAACGUGUCACGUTT |

**The partial sequences for ADAM17 plasmid**

| NO. | Primer sequence |
| --- | --- |
| plasmid | GACGGATCGGGAGATCTCCCGATCCCCTATGGTG |

**Supplementary Table 3.**

**The shRNA sequences targeting ADAM17**

| NO. | Primer sequence |
| --- | --- |
| ShADAM17 | CCCTTGAAGAATACTTGTAAA |

**Supplementary Table 4.**

**Primer sequence (human)**

| Gene | Primer sequence | Application |
| --- | --- | --- |
| ACSL4 | Forward Primer: ATGTCTGCTTCTGCTGCCCAAT | RT-qPCR |
|  | Reverse Primer: GGTGCTCCAACTCTGCCAGTAG |  |
| PTGS2 | Forward Primer: CCAGCACTTCACGCATCAGTT | RT-qPCR |
|  | Reverse Primer: TGTCTAGCCAGAGTTTCACCGTAA |  |
| GAPDH | Forward Primer: TGCACCACCAACTGCTTAGC | RT-qPCR |
|  | Reverse Primer: GGCATGGACTGTGGTCATGAG |  |
| GPX4 | Forward Primer: CCGCTGTGGAAGTGGATGAAG | RT-qPCR |
|  | Reverse Primer: CTTGTCGATGAGGAACTTGGTGAA |  |
| α-SMA | Forward Primer：TTGTACCGAACACAGGAAAGAAG | RT-qPCR |
|  | Reverse Primer：CCCAGCCATAGATGGTTGGTC |  |
| Fibronectin | Forward Primer：GACCAAGGAAATCGGCCTCTA | RT-qPCR |
|  | Reverse Primer：ACCTCTAGGCTGGCTATCTT |  |
| Collagen I | Forward Primer：TGAGCTCGCCAGTGAAATGA | RT-qPCR |
|  | Reverse Primer：AGATTCGTAGCTGGATGCCG |  |
| ADAM17 | Forward Primer：GCGGTGACCACGAGAATAATAAG | RT-qPCR |
|  | Reverse Primer：TCACACTCCTCTCCTTCATCCA |  |
